# A Pilot Adaptive Neurofeedback Investigation of the Neural Mechanisms of Implicit Emotion Regulation Among Women With PTSD

**DOI:** 10.3389/fnsys.2020.00040

**Published:** 2020-07-03

**Authors:** Shelby S. Weaver, Rasmus M. Birn, Josh M. Cisler

**Affiliations:** Department of Psychiatry, The University of Wisconsin—Madison, Madison, WI, United States

**Keywords:** adaptive neurofeedback, real-time fMRI neurofeedback, PTSD, emotion regulation, attention, interpersonal violence

## Abstract

Posttraumatic stress disorder (PTSD) is widely associated with deficits in implicit emotion regulation. Recently, adaptive fMRI neurofeedback (A-NF) has been developed as a methodology that offers a unique probe of brain networks that mediate implicit emotion regulation and their impairment in PTSD. We designed an A-NF paradigm in which difficulty of an emotional conflict task (i.e., embedding trauma distractors onto a neutral target stimulus) was controlled by a whole-brain classifier trained to differentiate attention to the trauma distractor vs. target. We exploited this methodology to test whether PTSD was associated with: (1) an altered brain state that differentiates attention towards vs. away from trauma cues; and (2) an altered ability to use concurrent feedback about brain states during an implicit emotion regulation task. Adult women with a current diagnosis of PTSD (*n* = 10) and healthy control (*n* = 9) women participated in this task during 3T fMRI. During two initial non-feedback runs used to train a whole-brain classifier, we observed: (1) poorer attention performance in PTSD; and (2) a linear relationship between brain state discrimination and attention performance, which was significantly attenuated among the PTSD group when the task contained trauma cues. During the A-NF phase, the PTSD group demonstrated poorer ability to regulate brain states as per attention instructions, and this poorer ability was related to PTSD symptom severity. Further, PTSD was associated with the heightened encoding of feedback in the insula and hippocampus. These results suggest a novel understanding of whole-brain states and their regulation that underlie emotion regulation deficits in PTSD.

## Introduction

A hallmark symptom of posttraumatic stress disorder (PTSD) is hypervigilance towards threats. Attentional bias towards threat is not only part of the diagnostic criteria for PTSD, but it is also a predominant focus of neurocircuitry models of PTSD. That is, prominent neurocircuitry models of PTSD (Rauch et al., 2006; Pitman et al., 2012; Admon et al., 2013) converge on the explanation that biased attention towards threat is mediated jointly by the hyperactive amygdala and dorsal anterior cingulate cortex (dACC) and hypoactive medial prefrontal cortex (mPFC). These PTSD neurocircuitry conceptualizations of attentional bias towards threat correspond with more general models of implicit emotion regulation (Etkin et al., 2006, 2015), which differentiate two processes engaged during attentional biases: the detection of a salient cue and a regulatory process by which attention is disengaged from the cue and redirected towards task-relevant stimuli. From this perspective, PTSD is characterized by heightened detection of trauma-related cues and weaker engagement of regulatory processes. Presumably, these are mediated by amygdala hyperactivity resulting in the capturing of attention, and rostral anterior cingulate cortex (rACC) hypoactivity failing to inhibit attention towards the trauma cue and redirect attention towards on-going task-relevant stimuli. The purpose of the current study was to use a novel methodology to provide a critical test of this neurocircuitry model of PTSD.

A significant limitation of current neurocircuitry models of implicit emotion regulation and PTSD is the univariate focus on isolated regions. By contrast, human neuroimaging is moving towards more plausible neurophysiological models, where the brain is conceptualized as a spatially distributed and multivariate network of regions operating in tandem (Bullmore and Sporns, 2009; Rubinov and Sporns, 2010; Smith, 2012; Sporns and Betzel, 2016; Woo et al., 2017). Multivariate pattern analysis (MVPA) is an analytic approach that offers a unique characterization of large-scale functional brain organization (Haynes, 2015). In this approach, a mapping is tested between a multivariate set of features (e.g., voxels) and cognitive states (e.g., attention to faces or words). When applying an MVPA approach to identify neural mechanisms of attentional bias towards threat in PTSD, there are at least two clear predictions. First, given the poorer behavioral performance on attentional bias tasks involving a threat in PTSD, a parallel brain representation might be expected. That is if attention is drawn to threat cues when directed to ignore threat cues (e.g., the common emotional Stroop task), underlying brain representations that encode “attend to threat” and “ignore threat” would be expected to be more similar in PTSD. Therefore, brain states would more poorly discriminate these distinct cognitive states due to an inability to suppress a brain state related to the distractor in those with PTSD compared to controls. Second, the distinct spatial patterns that discriminate these cognitive states should differ in PTSD vs. controls. Specifically, regions involved in salience detection (e.g., amygdala, insula, dACC) should have stronger contributions to the “attend threat” state in PTSD, whereas the rACC should have weaker contributions to the “ignore threat” state in PTSD. These predictions are directly testable using an MVPA approach.

Another interesting methodology that MVPA affords is in its application to real-time adaptive fMRI neurofeedback (A-NF), which involves the real-time presentation of a subjects’ blood-oxygen-level-dependent (BOLD) signal back to the participant (usually adapted into a moving scale or other forms of feedback) while they are in an MRI scanner. Then, the experimenter can adapt the behavioral task in real-time, based on the BOLD response of the participant (deBettencourt et al., 2015; Mishra and Gazzaley, 2015; Sitaram et al., 2017). Classic applications of A-NF involve a “target region,” or the brain area of focus during neurofeedback, in which participants are instructed to either up- or down-regulate during a behavioral task (Linhartová et al., 2019). For example, participants may be instructed to increase amygdala activity in response to positive images and are shown their progress with a moving bar that moves up or down as amygdala activity increases or decreases, respectively. Various forms of A-NF have been studied in patients with PTSD, especially investigating patients’ ability to regulate amygdala activity, and demonstrate the feasibility of utilizing this novel task design for patients with PTSD and the capability of these patients to regulate amygdala activity (Gerin et al., 2016; Nicholson et al., 2017, 2018; Misaki et al., 2018; Zotev et al., 2018; Zweerings et al., 2018). In addition, research has demonstrated that A-NF can not only influence activity in the targeted feedback area (i.e., the amygdala) but may affect whole-brain modulation and connectivity (Misaki et al., 2018). These results suggest particular importance of the use of novel analysis measures, like MPVA, to measure large-scale functional brain organization.

In another study of A-NF, a support vector machine (SVM) classifier was adopted to discriminate (i.e., differentiate; not to be confused with the use of “discrimination” as a cognitive mechanism of neurofeedback; Gaume et al., 2016) between “brain states” reflecting two separate emotional states (i.e., tenderness and anguish), and was able to successfully classify these states based on distributed patterns of brain activity (Lorenzetti et al., 2018). Based on this methodology and relevant to hypervigilance towards threat in PTSD, the experimenter could create a contingency between task difficulty and the person’s brain state, such that a brain state more resembling the desired state (i.e., “ignore threat” brain state) is rewarded by making the next trial easier, whereas a brain state more resembling the undesired state (i.e., “attend to threat” brain state) is punished by making the next trial harder. This methodology has been successfully used in a neutral task differentiating attention from faces and scenes among healthy adults (deBettencourt et al., 2015). Here, we exploit this A-NF methodology in the context of an attentional bias task to test whether this methodology can be used to modify underlying difficulty among individuals with PTSD in engaging brain states related to attending to vs. attending away from threat. That is, we tested whether making task difficulty contingent on brain state engagement could increase engagement of the desired brain state in PTSD. If successful, this would suggest a novel and direct way of retraining the underlying patterns of brain activity that mediate biased attention to threat in PTSD. However, given general neurocognitive and self-regulation impairments in PTSD (Vasterling et al., 2002; Seligowski et al., 2015), PTSD might be characterized by a poorer ability to adaptively regulate brain states following the online feedback signal. Indeed, a recent neurofeedback study targeting the anterior cingulate cortex (ACC) among individuals across three training days found poorer performance in ACC regulation activity in PTSD compared to controls (Zweerings et al., 2018).

Accordingly, the current pilot study used a novel MVPA approach to critically evaluate large-scale neural network patterns that contribute to implicit emotion regulation difficulty in PTSD. We focused on addressing four research questions: (1) Is PTSD characterized by poorer large-scale brain state discrimination of attending to threat vs. attending away from the threat? (2) Is PTSD characterized by altered organization of a brain state that discriminates attention to vs. away from threat compared to controls? (3) Does neurofeedback “correct” the ability to engage desired brain states in PTSD? Alternatively, (4) is PTSD characterized generally by a poorer ability to use feedback to regulate brain states? Addressing these research questions would provide novel evidence regarding the large-scale network patterns associated with implicit emotion dysregulation in PTSD and provide novel targets for treatment.

## Materials and Methods

### Participants

Study approval was granted by the University of Wisconsin Institutional Review Board following the Declaration of Helsinki. All participants consented before participation. Inclusion criteria were female sex, ages 21–50, and either: (a) a current diagnosis of PTSD related to interpersonal violence (IPV) exposure; or (b) no current mental health diagnosis and no current psychotropic medications. Three participants were excluded from analyses due to scanner error, a neurological abnormality that precluded registration, and inability to follow task instructions, therefore final analyses included 10 PTSD and nine healthy control participants. Demographic information is included in Table 1.

**Table 1:** Demographic information.

Variable	PTSD group *n* = 10	Control group *n* = 9	*p*-value
Age	33.8 (8.8)	31.8 (7.3)	0.60
IQ	101.9 (18.9)	113.1 (23.2)	0.26
Working memory score	10.1 (2.1)	9.6 (3.3)	0.67
Ethnicity			
Caucasian (%)	80.0	88.9	
African American (%)	10.0	0.0	
Other (%)	10.0	11.1	
Current Depressive Disorder (%)	33.3	–	
Current Anxiety Disorder (%)	77.8	–	
Current psychotropic medications (%)	30.0	–	
DERS Total Score	91.5 (27.4)	37.8 (8.6)	<0.001
CAPS-5 Total Score	46.5 (13.7)	–	
CAPS-5 Avoidance	5.3 (1.3)	–	
CAPS-5 Hyperarousal	10.8 (4.3)	–	
CAPS-5 Re-experiencing	11.1 (4.4)	–	
CAPS-5 Negative Cognitions/Mood	19.3 (6.6)	–	

*Note: PTSD, Post-traumatic stress disorder; IQ, Intelligence quotient; DERS, Difficulties in Emotion Regulation Scale; CAPS-5, Clinician-Administered PTSD Scale for DSM-5*.

### Assessments

All participants completed a clinical interview and questionnaires to assess mental health, trauma exposure, and verbal intelligence quotient (IQ). The Receptive One-Word Picture Vocabulary Test, Fourth Edition (ROWPVT-4) was used to measure verbal IQ (Brownell, 2000). The Structured Clinical Interview for DSM-IV (SCID-IV) was used to assess current and past mental health symptoms (First et al., 2002). Trauma exposure and assaultive history were assessed using the trauma portion of the National Women’s Survey (NWS) and National Survey of Adolescents (NSA; Resnick et al., 1993; Kilpatrick et al., 2000, 2013). PTSD diagnosis and symptom severity were assessed for any participants endorsing trauma exposure using the Clinician-Administered PTSD Scale for DSM-IV, Past Month Version (CAPS-4; Blake et al., 1995). All participants included in the PTSD group met current criteria for PTSD, assessed by the CAPS.

### Implicit Emotion Regulation Task

The implicit emotion regulation task was modeled after a prior A-NF task (deBettencourt et al., 2015) and broader attentional bias to threat literature in PTSD and anxiety disorders (McNally et al., 1990; Etkin et al., 2006; Bar-Haim et al., 2007; Cisler et al., 2011; Etkin and Schatzberg, 2011). A block design was used as opposed to an event-related design, supported by evidence that the emotional Stroop effect produces greater behavioral impairment during block design (Cisler et al., 2011). The task was programmed and implemented with Neurobehavioral Systems Presentation software.

#### Training Runs

**Figure 1:**
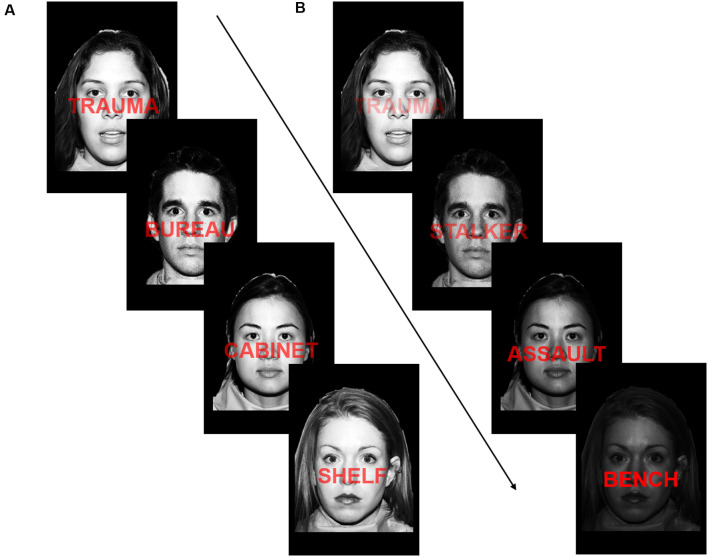
Depiction of the implicit emotion regulation task. **(A)** Example of the task during training runs. Face and word opacity remained static throughout training runs. **(B)** Example of the task during testing runs. On each trial, the opacity of the face or word changed in response to the degree to which a participant’s brain state matched the task instruction (“attend-to-word” or “attend-to-face”) on the previous repetition time (TR). Reproduced/Adapted from the NimStim Face Stimulus set with permission.

During the A-NF task training runs, words (either IPV-related or neutral) were embedded onto facial stimuli (either female or male). During the training phase, word and face opacity remained static throughout each run. Word categories were matched for syllables, length, and relative frequency of occurrence. Participants saw a series of images, each lasting 1s, presented on the screen. The instructions directed participants to attend to either the faces or words, in blocks of 30 trials lasting 1s each, and indicate (by button response) if the word (on attend-to-word blocks) was trauma-related or neutral and if the face (on attend-to-face blocks) was female or male. Ninety percentage of trials required a “yes” response, creating a prepotent tendency to respond “yes” (deBettencourt et al., 2015). Each block type was presented three times during training runs. Blocks were separated by an 8s resting-block with a fixation cross (Figure 1). Two training runs, used to train a SVM classifier SVM on the targeted brain states (details on SVM classification are provided below), used a factorial design with twelve blocks: six attend-to-face blocks (three with 90% neutral words, three with 90% trauma words) and six attend-to-word blocks (three with 90% neutral words, three with 90% trauma words). All blocks consisted of 90% female and 10% male faces from the NimStem Set of Facial Expressions (Tottenham et al., 2009).

#### Adaptive Neurofeedback Runs

The adaptive neurofeedback phase of the task consisted of two runs, each with six attend-to-face blocks and six attend-to-word blocks. All blocks used 90% trauma-related words. On each trial, the task difficulty was adjusted such that as the participants’ brain state moved away from the desired brain state, the opacity was adjusted to mask the target and make subsequent trials more difficult. For example, in an attend-to-face block, if a participant’s brain state moved closer to an attend-to-word brain state, the opacity of the faces became more transparent and words became dominant on the screen, making the faces harder to distinguish on future trials. Blocks were again separated by an 8 s resting block with a fixation cross.

#### MRI Acquisition

fMRI data were acquired on a GE MR750 3T scanner using an 8-channel headcoil. T1-weighted anatomic images were acquired with a MP-RAGE sequence [matrix = 256 × 256, 156 axial slices, repetition time/echo time/flip angle (TR/TE/FA) = 8.2 ms/3.2 ms/12°, field of view (FOV) = 25.6 cm, final resolution = 1 × 1 × 1 mm]. Echo planar imaging (EPI) sequences used to collect the functional images used the following parameters: TR/TE/FA = 2000 ms/25 ms/60°, FOV = 24 cm, matrix = 64 × 64, 40 sagittal slices, slice thickness = 4 mm, original resolution was 4 × 3.75 × 3.75 mm, and images were resampled to a final isotropic 3 × 3 × 3 mm.

### Real-Time fMRI Processing Pipeline

#### Classifier Training

Basic preprocessing was first applied to the initial two training runs and T1 images, all of which used AFNI software. The T1 image was skull stripped, registered to an image from the first training run, and segmented into gray matter, CSF, and white matter. The EPI images underwent slice time correction, deobliquing, motion correction, and 6 mm Full Width at Half Maximum (FWHM) spatial smoothing.

Following this basic preprocessing of the training data, the SVM classifier, using a radial basis function kernel and *C* = 1, was trained to differentiate attend-to-threat block (i.e., attend-to-word instructions with trauma words) TRs vs. ignore threat block (i.e., attend-to-face instructions with trauma words) TRs, and rest TRs were censored out. The input features consisted of z-scored time courses of all gray matter voxels, concatenated across both training runs. The training labels, corresponding to the attend-to-threat and ignore-threat blocks, were coded −1 and 1. The SVM classifier was then fit corresponding gray matter voxel time courses, using LIBSVM, to optimally define the hyperplane that differentiates the training labels. The resulting weights of each feature were then transformed into 3D spatial maps for further group-level analysis (see below). We conducted additional offline classification analyses (see below) using leave-one-out cross-validation to define the accuracy of the SVM classifier, but the classifier that was used during the adaptive neurofeedback phase used all available TRs to maximize power and accuracy of the classifier.

#### Adaptive Neurofeedback

After training the SVM classifier, the adaptive real-time fMRI neurofeedback runs began. The MRI console computer sent each TR through a Transmission Control Protocol/Internet Protocol (TCP/IP) connection to a separate Linux-based computer running AFNI’s real-time fMRI plug-in, which assembled and wrote each 3D volume to disk as it was acquired. The volume was registered to the training data registration image and 6 mm FWHM smoothing was applied. A Matlab script was concurrently running, which identified and loaded each volume after it was written, reshaped the volume to a 1D vector, and applied a gray matter mask. The task began with 20 resting-state TRs (40 s) to allow a sufficient sample with which to z-score and detrend the gray matter voxel time courses of the testing data before applying the SVM classifier, again using LIBSVM. After 20 TRs and applying z-scoring and detrending, the SVM classifier was fit to the current volume, resulting in a hyperplane prediction. This hyperplane prediction then first underwent a sigmoidal transformation and then was scaled to a range of 0.17–0.83 (i.e., the minimum stimulus opacity was 17% and the maximum was 83%, thus ensuring that both stimuli (face or word) were visible to at least a slight degree). After the initial three stimuli scaled sigmoidal transformed values, we applied a slight temporal smoothing to these values by taking the average of the current and previous two values. The current sigmoidal transformed and scaled hyperplane prediction was then transformed to the range of alpha values used by Neurobehavioral Systems Presentation software (0–255), which controlled the opacity of the different stimuli. These operations required between 800 ms and 1,200 ms, depending on the amount of data requiring z-scoring and detrending and was well-within the TR acquisition time of 2,000 ms. The current stimulus opacity value was then written to disk and a concurrently running python script transmitted it *via* TCP/IP connection to the stimulus presentation computer. The Neurobehavioral Systems Presentation software script running on the stimulus presentation computer then adjusted the alpha channel of the subsequent stimulus pair according to the incoming opacity value. That is, the subsequent trial displayed the opacity of each stimulus in direct accordance with the previous transformed hyperplane value. Opacities of both face and word stimuli were accordingly changed on a TR-by-TR basis in opposing directions, corresponding to the hyperplane prediction. This resulted in the stimulus opacity changing every two trials (i.e., as per the TR acquisition time). Each block began with balanced opacity (i.e., 50% face and 50% word) on the first two trials, and updated accordingly for the remainder of the trials in the block.

### Offline Preprocessing

Image preprocessing followed standard steps and was completed using AFNI software in the following order. Images underwent de-spiking, slice timing correction, deobliquing, motion correction using rigid body alignment, alignment to participant’s normalized anatomical images, spatial smoothing to achieve an isotropic smoothness of 8 mm (AFNIs 3dBlurToFWHM that estimates the amount of smoothing to add to each dataset to result in the desired level of final smoothing), detrending, low frequency (128 s) bandpass filtering, and rescaling into percent signal change. Images were normalized using the MNI 452 template brain. Following recent recommendations (Siegel et al., 2014), we corrected for head motion-related signal artifacts by using motion regressors derived from Volterra expansion, consisting of [R R^2^ R_t−1_ R^2^_t−1_], where R refers to each of the six motion parameters, and separate regressors for mean signal in the CSF and WM. This step was implemented directly after motion correction and normalization of the EPI images in the image preprocessing stream. Additionally, we censored TRs from the first-level generalized linear models (GLMs) based on the threshold of framewise displacement (FD) >0.4 mm. FD refers to the sum of the absolute value of temporal differences across the six motion parameters; thus, a cut-off of 0.4 mm results in censoring TRs where the participant moved, in total across the six parameters, more than ~0.4 mm plus the immediately following TR (to account for delayed effects of motion artifact). Additionally, we censored isolated TRs where the preceding and following TRs were censored, and we censored entire runs if 50% or more of TRs within that run were censored. No participants were removed with this criterion.

## Data Analysis

All linear mixed-effects (LME) models were conducted using Matlab’s fitlme function.

### Behavioral Performance (Training Runs)

We tested for group differences in behavioral performance during the training runs using a Group (PTSD = 1 vs. control= −1) * Attention (attend-to-word = −1 vs. attend-to-face = 1) * Valence (trauma words = 1 vs. neutral words = −1) * Run (run 1 vs. run 2) LME model on the performance measure of sensitivity. Consistent with prior research, sensitivity was used as the behavioral measure of interest as it indexes attention to the correct stimulus during the most difficult portion of the task (i.e., inhibiting a prepotent “go” response upon correct identification of a low-frequency “no-go” stimulus). Age and Education were added as covariates in all models, and all models estimated a separate by-subject random intercept and by-subject random slope for any within-subject variables (Attention and Valence). Therefore, the final model was defined as such:

Sensitivity ~ Attention * Valence * Group + Age + Education + (Attention*Valence|sub)

### Classifier Accuracy (Training Runs)

We conducted additional offline analyses to define the accuracy of each participant’s SVM classifier that was used during the adaptive neurofeedback phase. Here, classifier accuracy was defined using a leave-one-out cross-validation approach, in which one block of each attend-to-word and attend-to-face block was selected as hold-out sample TRs for testing and the SVM classifier was trained on the remaining blocks, again using z-scored time courses of gray matter voxels as input features as described above in “Classifier Training” section. The classifier defined on the training set was then applied to labels from TRs in the held-out test blocks and accuracy (% correct) was stored. This process was repeated until each block was used as the held-out test sample, and classifier accuracy was defined as mean % accuracy across all folds of the cross-validation. This process was repeated identically for attending-to-word and attending-to-face discrimination when the word was trauma-related (i.e., defining classifier accuracy in the presence of threat words) and when the word was neutral (i.e., defining classifier accuracy in the presence of neutral words). Group differences in brain state discrimination during the training runs were tested using these SVM Classifier accuracies as the dependent measure with a Group * Valence LME model to investigate the degree to which voxel-wise patterns of brain activation discriminate between Attention, and whether this is moderated by Valence. Age and Education were again included as covariates, and a random intercept and slope were included for Valence.

### Classifier Accuracy Predicting Behavioral Performance (Training Runs)

An additional LME model tested whether SVM Classifier accuracy during training predicted behavioral performance and whether this was moderated by PTSD. The LME model consisted of a Group * SVM Classifier accuracy * Valence factorial design predicting sensitivity. Age and Education were again added as covariates, with a random intercept and slope included for Valence, SVM Classifier accuracy, and their interaction.

### Group Differences in Classifier Organization

We tested whether PTSD was associated with a unique pattern of brain activation that discriminated between Attention and whether this was moderated by Valence. This was tested by comparing the groups on voxel-wise feature weight loadings from the SVM Classifier. Given that SVM feature weights represent a backward encoding model, it is necessary to transform them into a forward encoding model to interpret the weights for the task (Haufe et al., 2014; Haynes, 2015). We used a previously described model to create these transformed forward encoding maps (Haufe et al., 2014), which were then compared using voxel-wise LME models with factors for Group and Valence with covariates for Age and Education. The voxel-wise analysis was constrained within a group-level gray matter mask. Cluster-level thresholding, using contemporary methodology with an autocorrelated function (Eklund et al., 2016; Cox et al., 2017), corrected for whole-brain comparison, in which a corrected *p* < 0.05 was achieved through 13 or more contiguous voxels (nearest neighbor = 1) with an uncorrected *p* < 0.001.

### Brain State Regulation (Neurofeedback Runs)

Brain state regulation was defined by correspondence between observed brain state and instructed brain state. During the A-NF phase, the participant-specific SVM Classifier was applied to each TR, resulting in a hyperplane distance (HD) quantifying the degree to which the current brain state resembles the brain state encoding the training labels. For example, with attend-to-face (= 1) and attend-to-word (= −1) to train the classifier, a TR with a more positive HD during testing reflects a brain state more closely matching the brain state the participant was using to attend to faces during training, whereas a TR with a more negative HD during testing reflects a brain state that more closely resembles the brain state the participant used to attend to words during training. Standard GLMs then regressed these time courses of HDs onto a task design matrix, consisting of two distinct block regressors for the Attention instructions and convolved with the standard HRF from SPM. The resulting beta coefficients represent the fit (i.e., correspondence) between the instructed brain state and the observed brain state (e.g., a higher beta coefficient suggests the HD corresponds better with the task design). These beta coefficients were then carried forward to a group-level Group*Attention LME model. In the group-level LME model brain state was regressed on Group, Attention, Run and their interaction, with Age and Education, included as covariates. A random intercept and slope were included for each within-subjects variable (Run and Attention) and their interaction.

### Brain Regions Encoding Neurofeedback

We compared groups on neural mechanisms encoding the A-NF signal by including the stimulus opacities, determined by the participant’s brain state on the previous trials, as additional predictors of the task design matrix of the standard, within-subject-GLMs. This task design included a column for attend-to-word and attend-to-face instructions, the attend-to-word instructions × word opacities, and the attend-to-face instructions × face opacities. These design matrices were created and implemented using AFNI (3dDeconvolve and 3dREMLfit). The beta coefficients, reflecting the degree to which trial-by-trial fluctuations in stimulus opacity explain variance in a given voxel’s activity, were then carried forward to group-level Group*Attention LME models, implemented voxel-wise within a group-level gray matter mask. Cluster-level thresholding (Eklund et al., 2016; Cox et al., 2017) corrected for whole-brain comparison, in which a corrected *p* < 0.05 was achieved through 13 or more contiguous voxels (nearest neighbor = 1) with an uncorrected *p* < 0.001.

## Results

### Behavioral Performance (Training Runs)

**Figure 2:**
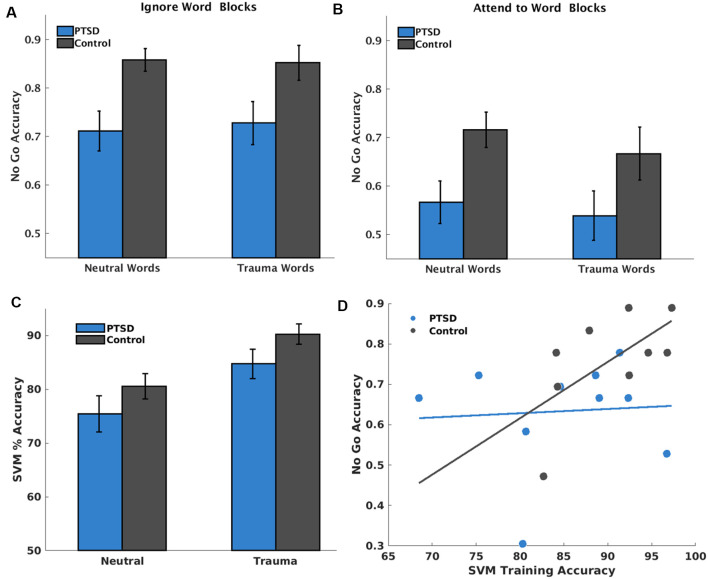
Behavioral performance and classifier accuracy during training runs. **(A)** Mean No Go accuracy on blocks where participants were instructed to ignore the words (i.e., attend to faces), separated by whether the block included trauma or neutral words. **(B)** The same as **(A)** except for blocks where participants were instructed to attend to the words. **(C)** Group differences in support vector machine (SVM) classifier accuracy during training runs in attend-to-word and attend-to-face blocks dependent on the presence of trauma-related words. Results showed significantly poorer classifier accuracy during neutral word blocks for both posttraumatic stress disorder (PTSD) and control groups, but no overall group differences on classifier accuracy. **(D)** Scatterplot depicting the significant linear relationship between mean SVM cross-validation accuracy and mean no go accuracy, suggesting that better brain state differentiation is related to better attention towards task-relevant stimuli.

The LME model identified a significant main effect of Attention, *t*_(134)_ = −5.66, *p* < 0.001, and group, *t*_(134)_ = −3.57, *p* < 0.001, on sensitivity during training runs. However, the main effect of Group was qualified by a Run*Group interaction, *t*_(134)_ = −2.04, *p* = 0.043, such that participants with PTSD showed decreased sensitivity during training Run 2 compared to controls, *t*_(66)_ = −3.52, *p* < 0.001, and a less pronounced decrease during Run 1, *t*_(66)_ = −1.88, *p* = 0.065. There were no other significant interactions (Figures 2A,B).

### Classifier Accuracy (Training Runs)

The LME model identified a main effect of Valence, *t_(_*_32)_ = 4.11, *p* < 0.001, on SVM Classifier accuracy during training runs, but no significant main effect of Group, *t*_(32)_ = −1.03, *p* = 0.312, or Group*Valence interaction, *t*_(32)_ = −0.074, *p* = 0.942 (Figure 2C).

### Classifier Accuracy Predicting Behavioral Performance (Training Runs)

The LME model identified a significant Valence*Group*SVM Classifier accuracy interaction, *t*_(28)_ = −3.05, *p* = 0.005, predicting sensitivity, specifically attributable to a Group*SVM Classifier accuracy interaction, *t*_(13)_ = −2.61, *p* = 0.022, during trauma, but not neutral blocks (*p* = 0.08). In both groups, higher SVM Classifier accuracy predicted better sensitivity; however, this effect was much stronger in the controls, *t*_(5)_ = 6.69, *p* = 0.001, than in the PTSD group, *t*_(6)_ = 3.88, *p* = 0.008 (Figure 2D).

### Group Differences in Classifier Organization

**Figure 3:**
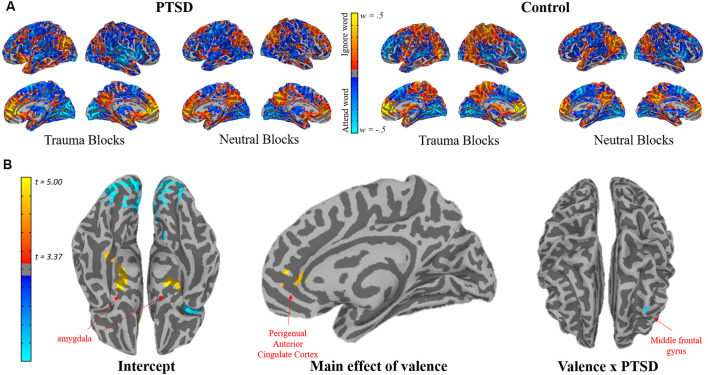
**(A)** Surface renderings of the mean SVM feature weights for the PTSD (left) and control (right) groups, separated by blocks containing trauma words vs. neutral words. The SVM classifiers were trained to differentiate attention towards the word target vs. attention towards the face target (i.e., ignoring the word target). Each participant’s raw SVM feature map underwent a forward encoding transformation before calculating group means to allow the maps to be interpretable concerning the brain processes of interest (Haufe et al., 2014). **(B)** Significant clusters of activation from a group-level linear mixed-effects (LME) model, in which a given voxel’s feature weight is modeled as a function of valence (trauma vs. neutral word blocks) × group (PTSD vs. control), with additional covariates for age and education. The intercept (bottom left) represents the mean feature weight encoding of the voxel, independent of factors entered into the model. The main effect of valence (bottom middle) indicates a significant cluster of voxels in the medial prefrontal cortex that more strongly differentiated attention to words vs. faces when trauma words were present. The valence × PTSD interaction (bottom right) indicates a significant cluster where the impact of valence on the differentiation of attention to words vs. faces differed between PTSD and control participants.

**Figure 4:**
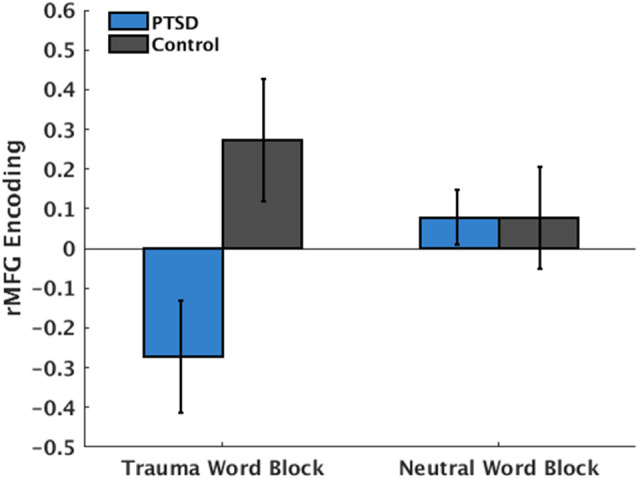
PTSD × Valence interaction in the right middle frontal gyrus (rMFG). More negative values indicate the heightened encoding of words during the block. More positive values indicate the heightened encoding of faces during the block. Individuals with PTSD showed heightened encoding of words only when word content was trauma-related but encoded faces when word content was neutral. Control participants showed heightened encoding of faces, regardless of word content.

The mean participant SVM maps the following transformation into forward encoding maps (Haufe et al., 2014) are depicted in Figure 3A. Results from the voxel-wise LME models on these forward encoding maps are depicted in Figure 3B and Table 2. Across participants, there was a main effect of Valence (i.e., discriminating between attending-to-words vs. faces during trauma vs. neutral blocks) in the perigenual anterior cingulate cortex (pgACC), indicating greater pgACC engaging when participants were instructed to ignore trauma words. There was a Group * Valence interaction in the right middle frontal gyrus (rMFG), indicating that during trauma-word blocks, the rMFG strongly encoded-words in individuals with PTSD, but strongly encoded faces in controls. During the neutral-word blocks, the rMFG encoded faces in both the PTSD and the control groups. Therefore, the PTSD group showed a marked sensitivity to Valence and strongly encoded-words only when they were trauma-related. In contrast, controls did not show a sensitivity to Valence, and more strongly encoded faces across all task blocks (Figure 4). To test the functional relevance of this region, Sensitivity was included as a covariate predicting rMFG encoding. An interaction between Valence and Sensitivity, *t*_(31)_ = 6.57, *p* < 0.001, indicated that greater rMFG encoding of faces during trauma blocks was associated with better Sensitivity, *t*_(14)_ = 2.16, *p* = 0.049.

**Table 2:** Results from the voxel-wise LMEMs on forward encoding maps.

		MNI center-of-mass coordinates				
Contrast	Region	*X*	*Y*	*Z*	Peak *t*	Cluster size
Intercept	Occipital Cortex	−2	83	6	−10.5	1,218
	Medial Prefrontal Cortex	−3	−46	6	10.4	423
	Right Anterior Insula	−43	−18	3	−5.5	157
	Superior Temporal Gyrus	−52	22	−4	−4.4	63
	Amygdala	20	9	−14	5.3	54
	Supplementary Motor Area	−6	−7	59	−4.7	54
	Inferior Frontal Gyrus	−26	−33	−5	6.9	46
	Amygdala	−23	1	−14	5.1	40
	Inferior Temporal Gyrus	30	28	−27	5.7	19
	Inferior Frontal Gyrus	23	−35	−5	4.5	18
Valence	Medial Prefrontal Cortex	−8	−41	4	4.5	26
Valence × PTSD	Middle Frontal Gyrus	29	−27	55	−4.7	14

### Brain State Regulation During Neurofeedback

**Figure 5:**
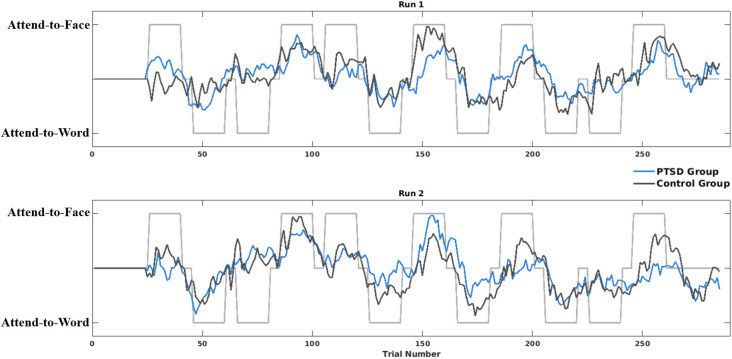
Hyperplane distance (HD) of both PTSD and control groups across all trials of both A-NF runs, plotted over the task labels (“attend-to-face” = 1, “attend-to-word” = −1). HD values quantify the degree to which the individual’s current brain state resembles the brain state encoding the training labels (based on training runs). Therefore, HDs more closely matching the task label reflect brain states more closely matching the brain state used to train the SVM classifier.

**Figure 6:**
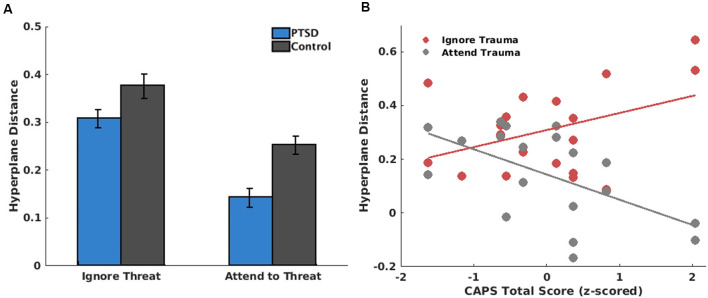
**(A)** Comparison of β coefficients for brain state regulation between groups as a function of task instruction. β coefficients come from generalized linear models (GLMs) in which HDs (i.e., SVM model predictions) were regressed onto the task design matrix. A higher β coefficient indicates better brain state regulation (i.e., the participant increased or decreased the brain state following task instructions). **(B)** Scatter plot indicating a significant PTSD symptom severity (CAPS total score) × task instruction interaction on β coefficients among the PTSD participants. As can be seen, more severe PTSD symptoms were associated with better brain state regulation when the task instruction was consistent with avoidance (i.e., ignore trauma words), yet worse brain state regulation when the task instruction asked participants to attend to the trauma words.

The LME model on brain state regulation during A-NF demonstrated significant main effects for Attention, *t*_(62)_ = −2.64, *p* = 0.011, and Group, *t*_(62)_ = −3.41, *p* = 0.001. However, this main effect of Group was again qualified by a Run*Group interaction, *t*_(62)_ = −2.06, *p* = 0.044, where participants with PTSD had particularly worse brain state discrimination (i.e., smaller hyperplane distance) during Run 2 compared to controls, *t*_(30)_ = −4.71, *p* < 0.001 (Figure 5). There were no other significant higher-order interactions (Figure 6A). Differences in PTSD symptom severity (CAPS total score) relating to brain state regulation performance during A-NF were then tested. This analysis consisted of an Attention*CAPS LME model controlling for Age and Education and identified a significant Attention*CAPS interaction, *t*_(32)_ = −3.17, *p* = 0.0034. This interaction was due to a positive association between CAPS severity and brain state regulation when the instruction was to avoid the trauma word, but a negative association when the instruction was to attend to the trauma word (Figure 6B).

### Brain Regions Encoding Neurofeedback

**Figure 7:**
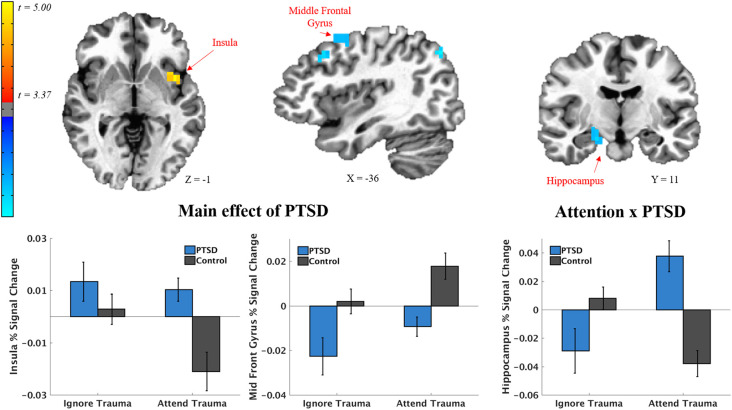
Significant clusters from group-level LME models of adaptive neurofeedback encoding where there was a main effect of PTSD (left) or interaction (right) with PTSD and attention instruction (attend-to-word vs. ignore word). Adaptive neurofeedback encoding refers to brain region activity that scales linearly with the feedback signal itself. In this paradigm, the feedback signal was the alpha channel (i.e., opacity) of the target stimulus. The feedback signal updated on a trial-by-trial basis depending on the participant’s hyperplane, which itself was determined through the fit of the participant’s brain state on a given trial with the SVM classifier. Higher alpha channels of the target stimulus indicate positive feedback, such that they inherently make the task easier and therefore reinforce brain states that preceded them. Below each significant cluster is a bar graph indicating mean activation differences per task condition as a function of PTSD.

Results of the voxel-wise LME model testing group differences in encoding trial-by-trial fluctuations in the A-NF signal (i.e., the opacity of target) are depicted in Figure 7 and Table 3. There was increased encoding of the A-NF signal in the anterior-to-mid insular cortex in PTSD participants and decreased encoding in two clusters in the MFG and one cluster in the parietal cortex. Further, there was an Attention*Group interaction in the hippocampus, such that in attend-to-word blocks (where 90% of words are trauma-related), there was a negative relationship between the A-NF signal and hippocampal activation in the control group (i.e., hippocampal activity tracked A-NF signals indicating poorer performance), but a positive relationship in the PTSD group (i.e., hippocampal activity tracked A-NF signals indicating better performance).

**Table 3:** Results from the voxelwise LMEM testing group differences in encoding trial-by-trial fluctuations.

		MNI center-of-mass coordinates				
Contrast	Region	*X*	*Y*	*Z*	Peak *t*	Cluster size
PTSD	Middle Frontal Gyrus	−40	−6	53	−5.2	43
	Occipital Cortex	−11	79	44	−5.5	28
	Insula	37	−6	−1	4.5	13
	Dorsolateral Prefrontal Cortex	−37	−20	40	−4.5	13
	Occipital Cortex	−35	68	42	−4.8	13
Attention × PTSD	Cerebellum	−13	37	54	−4.6	30
	Hippocampus	−20	10	−13	−4.1	13

## Discussion

The current pilot study used a novel task and MVPA adaptive neurofeedback approach to critically evaluate the large-scale neurocircuitry patterns mediating implicit emotion regulation in PTSD and whether A-NF can “correct” the ability to engage in desired brain states. During training runs of the attentional bias task, participants with PTSD showed reduced behavioral performance compared to controls, especially during run 2. During testing runs, when incorporating trial-by-trial feedback was necessary for task performance, participants with PTSD were also less able to incorporate relevant feedback, leading to reduced discrimination between brain states compared to controls, particularly during the second testing run. In addition, SVM classifier accuracy was positively related to task accuracy across participants, such that as the SVM classifier became more accurate (i.e., brain states for the target vs. distractor were more easily discriminable), participants’ task performance increased. This suggests that performance on an attentional bias task may at least in part require the ability to effectively engage different brain states and suppress a brain state related to the distractor. Interestingly, this relationship between SVM Classifier accuracy and behavioral performance was significantly weaker in individuals with PTSD compared to controls only during the trauma word blocks, suggesting this impairment may be specific to a trauma-related context. Therefore, even without overall differences in SVM classifier accuracy, these individuals may not as effectively “switch” into brain states that increase task performance during blocks involving trauma-related stimuli. While these results did not support the hypothesis that individuals with PTSD would be overall less able to discriminate between brain states, we did find evidence that brain state regulation in PTSD was less strongly related to task performance when trauma-stimuli were presented.

The current study did not find support for the typical neurocircuitry model of PTSD (which would predict increased amygdala activity couple with decreased frontal activity). However, these results do suggest an impairment may be due to deficits in the ability to switch attention. Seen in Figure 5, individuals with PTSD, especially during later blocks of the testing runs, show almost no discrimination between brain states in either attend-to-word or attend-to-face blocks. These individuals may effectively get “stuck” attending to the trauma-related words in each block, and therefore are unable to shift their attention to the relevant task instructions. In run 2, this effect may be exacerbated by the high cognitive demands of the behavioral task coupled with the repeated presentation of trauma-related stimuli, which differentially influence behavior in those with PTSD compared to healthy controls. These deficits in the switching of attention away from trauma-related cues and onto task-relevant demands are crucial targets for treatment, and coupled with neurofeedback could be an important target for therapies moving forward.

Evidence also partially supported the idea that a weakened ability to effectively engage brain states among those with PTSD would be driven by different spatial patterns during discrimination compared to controls. Contrary to previous neurocircuitry models of implicit emotion regulation in PTSD, discriminability of attention towards vs. away from threat does not seem to involve a hyperactive amygdala. Instead, during trauma-word blocks, PTSD participants showed heightened encoding of words in the rMFG, whereas controls showed stronger encoding of faces. During neutral-word blocks, both groups strongly encoded faces in the rMFG. The degree to which this region encoded faces during trauma blocks was predictive of better behavioral performance, demonstrating a functional impact of this altered encoding. This demonstrates a specific sensitivity to the valence of the blocks in those with PTSD, strongly encoding words in the rMFG only when they were trauma-related, and possibly a mechanism of poorer behavioral performance. Right MFG has been proposed as a link between the dorsal attention network, responsible for top-down attention, and the ventral attention network, responsible for bottom-up attention, making it responsible for the reorientation of attention from external cues to exert endogenous control (Corbetta et al., 2008; Japee et al., 2015). Heightened encoding of words in this region during trauma-word blocks may indicate increased difficulty in ignoring the trauma-related words among those with PTSD, leading to greater activity as the rMFG attempts to reorient attention. Finally, the pgACC was significantly engaged in both the PTSD and control groups when instructed to ignore trauma cues, consistent with its theorized role in implicit emotion regulation (Etkin et al., 2011; Marusak et al., 2016), but inconsistent with univariate dysfunction within this region in PTSD.

During testing runs involving ongoing neurofeedback in response to feedback signals (opacity), PTSD participants showed decreased brain state discrimination compared to controls, especially in run 2. As such, providing feedback signals about their brain state did not improve control over the ability to engage the desired brain state. Rather, consistent with prior research (Zweerings et al., 2018), PTSD was associated with poorer regulation of brain states during neurofeedback. This could be explained by a variety of factors. First, general neurocognitive and emotion-regulation impairments in PTSD could negatively impact their ability to respond and update brain states based on feedback signals. Participants with PTSD have been shown to perform significantly worse on tasks involving sustained attention, therefore cognitive demands of the attentional bias task could differentially affect PTSD participants compared to controls (Vasterling et al., 2002). However, PTSD participants in this sample had average intelligence and working memory abilities, suggesting baseline cognitive deficits were likely not responsible.

Second, this inability to regulate brain states could also depend on the context in which participants with PTSD are asked to discriminate. There was an observed interaction between PTSD symptom severity and brain state regulation during separate blocks of the A-NF task, such that in task blocks instructing participants to avoid attending to the trauma stimuli, participants with higher PTSD symptom severity showed increased brain state discrimination. That is, they were better able to use feedback signals specifically to suppress the brain state that encoded trauma words. However, during the attend-to-trauma task blocks, PTSD symptom severity showed a negative correlation with brain state discrimination, such that participants with higher PTSD symptoms were less able to discriminate between attend-to-trauma and avoid-trauma brain states. That is, they were worse at using feedback signals to engage brain states encoding trauma words. This context-dependent ability to regulate brain states is directly in line with the conceptual understandings of PTSD. Higher symptoms of PTSD would suggest a natural tendency to avoid trauma reminders, and participants with higher symptoms may be more effective in tasks that encourage this. However, when task instructions directly contradict this natural tendency (i.e., asking participants to attend to a trauma reminder), participants with higher symptoms would be less able to engage brain states that encode trauma words. Relevant to contemporary neurocircuitry models of PTSD, the current results also provide novel evidence that avoidance of trauma stimuli is mediated by a widely distributed and multivariate brain state, rather than being localized to a given region as normative and PTSD neurocircuitry models based on univariate analyses would suggest (e.g., mPFC; Pitman et al., 2012; Etkin et al., 2015).

Relevant for PTSD neurocircuitry models, PTSD participants showed increased encoding of the neurofeedback signal in the anterior-to-mid insular cortex and decreased encoding in the MFG and parietal cortex. This indicates that PTSD participants may be recruiting less typical networks responsible for top-down cognitive control (i.e., frontoparietal network), and instead recruit regions more involved in salience detection (Patel et al., 2012; Admon et al., 2013). In healthy subjects, previous studies have shown the insular cortex responds similarly to opposite neurofeedback signals (i.e., up and down-regulate amygdala), suggesting a lack of discrimination in this region to certain types of neurofeedback (Paret et al., 2018). Given these results, the increased encoding of neurofeedback signals seen in individuals with PTSD is especially notable. Instead of executing top-down control to incorporate feedback information on later trials, participants with PTSD seem to focus on the salience of that information instead. This inability to incorporate feedback could explain the inability to discriminate brain states during testing when feedback is critical for performance.

In addition to group differences in neurofeedback signal encoding, analyses revealed group differences in hippocampal activity during different instruction phases (attend-to-trauma, ignore trauma). For controls, hippocampal activity was related to the encoding of signals indicating poorer performance in the attend-to-trauma condition, whereas, in PTSD participants, hippocampal activity was related to encoding signals indicating better performance. This is in line with previous research suggesting those with higher PTSD symptoms perform better on reward-based feedback trials compared to punishment trials in cognitive tasks (Myers et al., 2013), and may also suggest an impairment in those with PTSD in incorporating negative feedback when in a context requiring attention to trauma-related stimuli. These data suggest that decreased discriminability between brain states may underlie hypervigilance towards threat cues in PTSD and lead to poorer behavioral performance when tasks require disengagement of attention from trauma cues (i.e., during trauma-word blocks). Although this pilot study suggests a novel way of understanding the neurobiological underpinnings of brain state regulation in those with PTSD, it is not without limitations. The use of a novel behavioral task in addition to small numbers of participants in each group makes replication crucial to the understanding of the neurocircuitry of PTSD. Given that participants with PTSD in this study show a weakened ability to engage brain states to increase performance, and show overall decreased task performance during training runs, it will be essential for future research to determine whether those with PTSD can improve this brain state regulation, as this could inform targeted therapy techniques in the future.

## Data Availability Statement

The datasets generated for this study are available on request to the corresponding author.

## Ethics Statement

The studies involving human participants were reviewed and approved by University of Wisconsin—Madison Institutional Review Board. The patients/participants provided their written informed consent to participate in this study.

## Author Contributions

SW was involved in study design, recruitment, analysis, interpretation and manuscript writing. RB was involved in study design and manuscript writing. JC was involved in study design, recruitment, analysis, interpretation and manuscript writing.

## Conflict of Interest

The authors declare that the research was conducted in the absence of any commercial or financial relationships that could be construed as a potential conflict of interest.
